# GPU-accelerated lung CT segmentation based on level sets and texture analysis

**DOI:** 10.1038/s41598-024-51452-6

**Published:** 2024-01-16

**Authors:** Daniel Reska, Marek Kretowski

**Affiliations:** Faculty of Computer Science, Bialystok University of Technology, Białystok, Poland

**Keywords:** Computer science, Image processing

## Abstract

This paper presents a novel semi-automatic method for lung segmentation in thoracic CT datasets. The fully three-dimensional algorithm is based on a level set representation of an active surface and integrates texture features to improve its robustness. The method’s performance is enhanced by the graphics processing unit (GPU) acceleration. The segmentation process starts with a manual initialisation of 2D contours on a few representative slices of the analysed volume. Next, the starting regions for the active surface are generated according to the probability maps of texture features. The active surface is then evolved to give the final segmentation result. The recent implementation employs features based on grey-level co-occurrence matrices and Gabor filters. The algorithm was evaluated on real medical imaging data from the LCTCS 2017 challenge. The results were also compared with the outcomes of other segmentation methods. The proposed approach provided high segmentation accuracy while offering very competitive performance.

## Introduction

Efficient and accurate medical image segmentation is one of the biggest challenges in modern computer vision^1,2^. Nowadays, this task is often an important step in the analysis of both two-dimensional (2D) images and three-dimensional (3D) imaging datasets. Although the typical imaging datasets contain 3D structures, many traditional segmentation methods are limited to processing of individual images in a “slice-by-slice” manner^2,3^. Such an approach can be prone to errors and tedious due to a large number of images in datasets. Fully 3D segmentation algorithms^4,5^ can offer clear improvements, eliminating the need for the processing of separate slices and providing natural continuity and smoothness of the segmented regions.

While the accuracy of the segmentation is the main factor in the medical context^3,5^, the time performance of the algorithms can also be a major issue, particularly due to the size of 3D imaging data^4^. The efficiency of the segmentation methods can greatly benefit from acceleration by graphics processing units (GPUs)^4,6,7^. GPUs are inherently well-equipped to handle parallel processing of 2D and 3D image data.

This research proposes a fully 3D semi-automatic method for lung CT segmentation. The algorithm adopts a texture-based level set formulation of an active surface model that is initialized using a probability-based scheme. The operator is required to place only a few (2 or 3) small ellipses inside the lungs. These initial regions are used to generate a starting form of the level set, according to the probability distribution of the texture features inside the regions. The level set-based implicit surface is then evolved up to the final segmentation result. The realised implementation integrates features based on grey-level co-occurrence matrices (GLCM)^8,9^ and Gabor filters^10^. The algorithm was evaluated on real medical imaging data and its results were compared with outcomes of other state-of-the-art segmentation methods. The proposed method provided high segmentation accuracy while offering very competitive performance thanks to GPU acceleration.

### Background and related works

The task of lung segmentation in CT datasets was always an important and competitive problem to solve^2,3^. The algorithms have to take into consideration issues like pathological lung tissue and the close neighbourhood of other organs. Normal anatomical structures in the hilum of the lung, like blood vessels, nerves, and lymph nodes, can also pose significant difficulties. The need for efficient lung segmentation also increased nowadays due to the consequences of the COVID-19 pandemic^11–13^. Over the years, many types of methods were developed to tackle the lung segmentation problem, like thresholding^14,15^, region growing^16^, watershed^17^ and atlas-based algorithms^18,19^. The currently popular methods also include deep learning (DL) methods^12,20,21^. Furthermore, a pipeline of multiple algorithms can be utilised^14,15,18^, where separate stages of the segmentation problem are solved with different methods.

Deformable models, based on an idea of a shape that adapts itself to a target image region, are another class of methods used in (lung) segmentation. Level set-based models are particularly popular and found applications e.g. in automatic extraction of lung boundary in 2D CT images^22^, nodule segmentation based on variational level sets and prior shape models^23^, or in segmentation in tandem with registration algorithms^24^. 3D methods often make use of discrete active models^25^ since level sets can be computationally intensive and require solutions with GPU acceleration^6,26^ to achieve acceptable performance.

The current state-of-the-art in lung segmentation is dominated by DL methods, but more traditional region- and shape-based methods are still being proposed^27^. In the case of DL, the quality and availability of annotated training data are often as important as the employed algorithmic methodology^28^. The process of labelling imaging data requires a skilled operator, can be time-consuming, and often relies on interactive and semi-automatic segmentation tools. Traditional methods like deformable models can be beneficial in interactive and semi-supervised tasks that have to deal with atypical cases, or when the availability of labelled data is limited^13^. Further development of semi-automatic solutions is therefore still valuable.

To improve the characterization of different lung tissues, image texture analysis is often applied to aid in lung segmentation^29^ and classification of lesions and nodules^30,31^. Although effective in these tasks, texture features are typically computationally intensive and therefore it can be difficult to efficiently integrate them with semi-automatic methods, even with the help of GPU acceleration^4^. In the case of level set-based active models, past GPU-accelerated methods relied on simple intensity features^32,33^, and more advanced image features posed a significant computational burden^26^. Fortunately, the capabilities of modern graphics cards make the integration of texture analysis with interactive methods a more feasible goal that is worth pursuing.

**Figure 1:**
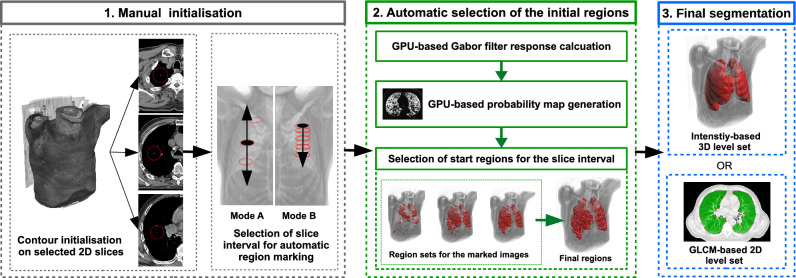
Diagram of the proposed method. The initial ellipses are placed on selected slices of the dataset. Determining the range of images for further analysis is possible by (Mode A) setting the interval whose center is the image with the selected contour or (Mode B) propagating the selected contour from the beginning of the lung area down the z-axis.

## Materials and methods

The proposed algorithm aims to provide a semi-automatic segmentation of the lung tissue with minimal involvement of the operator. The method (see Fig. 1) consists of three main steps:

- manual initialisation of a small number (typically from 2 to 3) of 2D contours inside the target lung area;
- automatic selection of starting regions for the active contour/surface model;
- evolution of the model using a level set formulation.

The main feature of the method is the efficient creation of the initial areas, which occupy a significant part of the target region (compared to typical manual initialization) as it enables faster evolution of the deformable model. The use of textural features is intended to improve the initialization and operation of the model by capturing the texture of the lung tissue, which facilitates the segmentation of their area and the exclusion of neighboring organs.

### Initialisation and starting region selection

**Figure 2:**
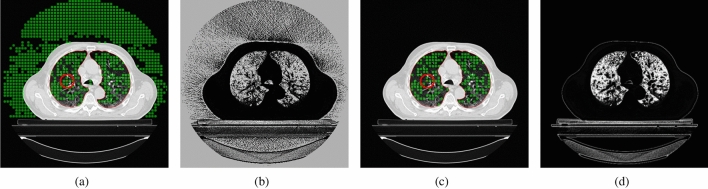
Initialization of the starting regions of the model based on the marked contour: (**a**) contour with areas generated based on image intensity, (**b**) probability map for intensity, (**c**) selected regions created with Gabor features, and (**d**) probability map based on Gabor features.

In the beginning, the algorithm requires the operator to place a few circular curves inside the lung area. These initial regions are used to generate a starting form of the level set, according to the probability distribution of the features inside the regions. Figure 1 shows an example initialisation with three contours placed inside the upper, middle, and lower lobes of the right lung. Next, for each slice with a contour *C*, a probability of inclusion into the region marked by *C* is calculated for all pixels (points) in the image. The inclusion probability $P_{in}$^34^ for a point *p* in the *I* image can be generated according to:

1 $$\begin{aligned} P_{in}(p,I,C) = \frac{1}{|V(C)|} \sum _{v \in V(C)} \frac{1}{\sqrt{2 \pi \sigma }} e^{\frac{-(I(p) - C(v))^2}{2 \sigma ^2}}, \end{aligned}$$

where *V*(*C*) is the set of pixels inside the *C* contour, *I*(*p*) and *C*(*v*) are the values of, respectively, the image *I* in the point *p* and inside the contour *C* in the point *v*. In this way, a map with the probability of each pixel in *I* belonging to the initial curve *C* can be created. This formulation operates only on the intensity of the image. The proposed approach incorporates a bank of Gabor features that were selected to capture the texture of the lung tissues in a more robust way (see Fig. 2). For each image, the used Gabor bank creates a set of feature maps $M_G$ that consist of the responses *m* of each specific Gabor filter. The final probability map $P_{tex}$ is a sum of the separate *m* maps for each of the generated features:

2 $$\begin{aligned} P_{tex}(p,M_G,C) = \sum _{m \in M_G} P_{in}(p,m,C). \end{aligned}$$

The probability maps are then utilised to generate the starting regions for the active surface method. First, a map for each image in the data set is partitioned into square regions (windows). Next, the similarity of each of the square regions is compared with the region of the closest initial contour (i.e. most of the images will not have a contour present, therefore the contour from the closest marked image in the series is selected). The similarity criterion checks if the difference in mean probability values in the window and contour areas does not exceed half of the standard deviation of the probability in the contour region. If this condition is met, the square window is marked and becomes an elemental start region for the next phase (see Fig. 2a,c). The utilisation of texture features eases the process of manual initialisation. The intensity alone has difficulties in differentiate the lung tissue from other regions of the image (please see Fig. 2a,b). Obtaining a satisfactory result without the use of Gabor filters would require a substantially bigger operator intervention.

Probability maps calculated in the above manner are created for all images that contain lung tissue. For most images without an own contour, the method selects the contour from the closest (in the z-axis) marked image. The developed method offers two modes of selecting the interval of images for analysis (see Fig. 1):

(A) selection by the given interval: $<n_s-r,n_s+r>$ for the marked image with the number $n_s$ and the range *r*, e.g. for $n_s = 60$ and the range $r=40$ images from 20 to 100 will be selected;
(B) selection from the starting image to the given range: $<n_s,n_s+r>$; in this mode, it is also possible to copy the original contour to subsequent images (e.g. to every 30th image in the range), which can limit the initialization only to one contour.

In both modes, an image spacing *m* can also be set, which will make only every *m*th image in the range to participate in the process. Finally, the regions generated on the stack serve as a starting form of a deformable model.

### Active model evolution

The prepared implementation can independently apply two level set-based deformable models: an intensity-based 3D active surface^32^ and a texture-based 2D model^35^ that utilises GLCM and Gabor features. Although the method is focused on 3D segmentation, the 2D model is also employed to emphasise the advantages of the fully three-dimensional approach.

An active surface *S* can be defined as a set of zero-level points $p=(x,y,z)$ of the function $\phi (p,t)$. This formulation gives $S=\{ p:\phi (p,t) = 0 \}$, where $\phi (p,t):\mathbb {R}^{4}\mapsto \mathbb {R}$ and *t* is the evolution time step. The surface evolves in its normal direction according to the following partial differential equation (PDE):

3 $$\begin{aligned} \frac{\partial \phi }{\partial t} = |\nabla \phi | F, \quad \phi (p,0)=\phi _0(p), \end{aligned}$$

where $\phi _0$ is the initial surface and *F* is a speed function *F*(*p*, *t*), which allows the surface to expand or contract in order to encompass the segmented region. In the proposed method, the surface is deformed using the speed function *F* that combines image and curvature terms for a point *p*:

4 $$\begin{aligned} F(p) = \alpha D(p) + (1-\alpha )\text {div}\bigg (\frac{\nabla \phi (p)}{|\nabla \phi (p)|}\bigg ), \end{aligned}$$

where *D*(*p*) is the image data term that drives the deformation, $\alpha \in [0,1]$ is a user-defined balancing parameter, and $\text {div}\big (\frac{\nabla \phi (p)}{|\nabla \phi (p)|}\big )$ is the surface curvature. The intensity-based version^32^ defines the data term as:

5 $$\begin{aligned} D(p)=\varepsilon -|I(p)-T|, \end{aligned}$$

where *I*(*p*) is the image intensity in *p*, while *T* is the intensity target (the mean intensity in the initial regions) and $\varepsilon$ is the tolerance. In the 2D case, the proposed method can also employ a multi-feature data term $D_{tex}(p)$  that takes into consideration the features (denoted as a set *M*) generated for the given volume (please refer to^35^ for the details on feature generation and selection). For each point *p*, a subset of features $S_p$ is defined as:

6 $$\begin{aligned} S_p = \big \{ m \in M: |m(p) - \bar{x}(m)| > \theta \cdot \sigma (m) \big \}, \end{aligned}$$

where $\bar{x}(m)$ and $\sigma (m)$ are the feature’s mean and standard deviation inside the initial regions, *m*(*p*) is the value of feature *m* in the point *p* and $\theta$ is a user-defined sensitivity parameter. The texture data term is then defined as:

7 $$\begin{aligned} D_{tex}(p)={\left\{ \begin{array}{ll} v &{} |S_p| = 0 \\ -v &{} \text {otherwise,} \end{array}\right. } \end{aligned}$$

where *v* is a predefined constant (equal to 2 by default to balance the curvature influence and to ensure numerical stability). Both terms encourage the model to expand into regions where the features are similar to the interior of the initial contour. Otherwise, the model is prompted to retract.

In both models, the level set equation is solved with a GPU-accelerated implementation that stops upon the convergence of $\phi$ or when a maximum number of iterations is reached. The convergence depends on the value of $\Delta \phi _{n}$, which is calculated as:

8 $$\begin{aligned} \Delta \phi _{n} = \bigm ||\phi ^{obj}_{n}| - |\phi ^{obj}_{n-j}| \bigm |, \end{aligned}$$

where $|\phi ^{obj}_{n}|$ is the number of voxels in the $\phi$ that are marked as the segmented object in the *n*-th iteration. In this way, the evolution can stop when there is no substantial change in the total volume of the segmented area in the previous *j* iterations.

### Datasets

The proposed solution was developed and tested on thoracic CT scans from the 2017 Lung CT Segmentation Challenge (LCTSC) database^36^. The LCTSC database contains scans of 60 subjects with the CT data in DICOM format, as well as ground truth segmentation of lungs and adjacent organs: heart, spinal cord and esophagus. The DICOM sets consist of a series of $512 \times 512$ images with the lungs present typically on 100 to 120 slices, with the total image count from 103 to 279. The scans were divides into three groups: 36 training datasets, 12 sets for offline competition task and 12 sets for online task, held at the 2017 Annual Meeting of American Association of Physicists in Medicine (AAPM)^20^.

The scans are publicly available at The Cancer Imaging Archive (TCIA)^37^. The data was used according to the TCIA Data Usage Policy and the experiments were carried out in accordance with relevant guidelines and regulations. The conducted research was performed solely on the public TCIA data and no additional human data or human participants were involved in the study.

### Implementation concerns

The proposed implementation is based on OpenCL^38^, a cross-platform framework for heterogeneous parallel programming, widely used for general-purpose computing on graphics processing units (GPGPU). The method is implemented in MESA^39^—a platform for the design and evaluation of deformable model-based segmentation methods. The implementation uses Java for the main logic and the C language for the GPU code in OpenCL.

GPU acceleration was crucial for high performance of the level set evolution, texture features calculation, and probability map generation. In the manual initialisation stage, the GPU-based methods operate on a single image, which is used to calculate current Gabor features and probability maps. After such an initialisation, the Gabor responses are generated for the entire volume to provide features for the initial region selection. In this case, the entire dataset is passed to the graphics card memory. The Gabor features for all images are calculated in a single pass of a GPU-based routine (kernel) using a convolution-based algorithm. The start regions are then created separately for each image in the slice range, where the bulk of the computations is performed by a kernel calculating the Gaussian probability. The final segmentation stage also relies heavily on the GPU, particularly in the case of the 3D level set algorithm. The level set PDE (see Eq. 3) is solved by a GPU-based iterative finite difference method, while the convergence criterion is checked on the CPU side. GPGPU implementation provides a significant speedup to the level set algorithm, which is consistent with other similar solutions^4^ and reduces the run time of the algorithm from minutes to just seconds, in comparison with a CPU-based implementation.

## Experimental evaluation

**Figure 3:**
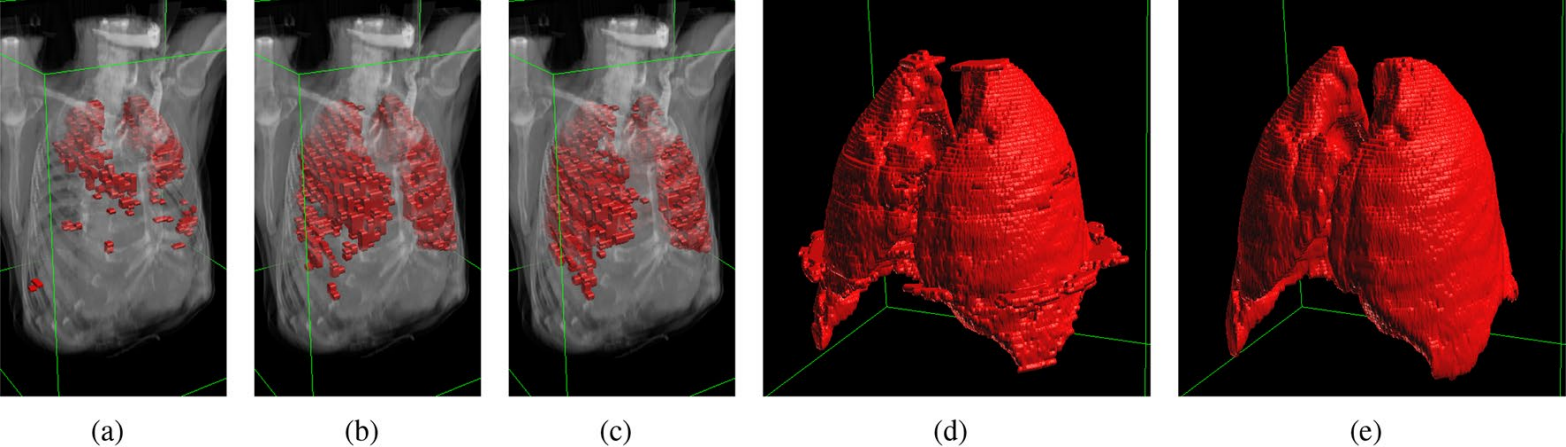
Visualisation of the initial active surface regions: the resulting regions from (**a**) upper, (**b**) middle, and (**c**) lower right lung lobes. Visual comparison of the results on LCTSC Train-S1-001 set obtained with: (**d**) 2D active contour, and (**e**) 3D active surface from the proposed method.

The proposed solution was evaluated on the LCTSC datasets. In the initialisation stage, the presented solution employed a bank of 6 Gabor filters with three orientations (0°, 45°, and 90°), wavelength of 8.0, two Gaussian envelope sizes (1.0 and 2.0), and envelope ratio of 0.25. During the experiments, the initial contours were manually placed inside the upper, middle, and lower lobes of the right lung (see Fig. 3). The 2D level set model utilized a set of GLCM features consisting of contrast, entropy, homogeneity, energy and correlation, with window radius up to 3 pixels and step size of 1. Unless otherwise specified, the default values of level set sensitivity ($\theta =4.0$, $\varepsilon =750$, $\alpha =0.0125$) and initial region radius of 5 pixels were used. The convergence of the 3D level set function was tested every 10 iterations and the volume change factor $|\phi ^{obj}_{n}|$ was set to about 0.5% of the voxel count of a typical target volume ($10^6$ voxels). The experiments were performed on a workstation with an Intel Xeon E5-1620v2 CPU, 16 GB RAM, and Nvidia Titan Xp GPU.

The proposed method was compared with other methods that competed in the AAPM Thoracic Auto-segmentation Challenge^20^, which processed the LCTSC database. The segmentation was performed according to the LCTSC guidelines (i.e. one lung should be separated from another and hilars and the trachea/main bronchus should be omitted).

### Segmentation quality validation

The quality of the segmentation was validated with multiple popular metrics^20,40^. The first three measures are *Dice coefficient* (DC), *volume overlap error* (VOE), and *relative volume difference* (RVD), defined as:

9 $$\begin{aligned} &\text {DC}= 2 \ |G \cap R| / (|G|+|R|), \\&\text {VOE}= 100 \ (1-|G \cap R| / |G \cup R| ),\\&\text {RVD}= 100 \ ((|R|-|G|)/|G|), \end{aligned}$$

where *R* is the set of result voxels, which is tested against the ”ground truth” reference segmentation *G*. These measures indicate the overlap and difference in volumes of the two regions. Additionally, two surface-based metrics were used: *mean surface distance* (MSD) and *95% Hausdorff distance* (HD95). MSD and HD95 take into account the distances between the surfaces of the segmentation and reference sets. With the surface voxel sets denoted as *S*(*G*) and *S*(*R*), MSD and HD can be defined as:

10 $$\begin{aligned} \text {MSD}(G,R)&=\frac{1}{|S(G)|+|S(R)|} \Bigg ( \sum _{s_G \in S(G)} d_S(s_G, S(R)) + \sum _{s_R \in S(R)} d_S(s_R, S(G)) \Bigg ), \\ \text {HD}(G,R)&= \max \Big \{ \sup _{g \in S(G)} \inf _{r \in S(R)} d(g,r), \sup _{r \in S(R)} \inf _{g \in S(G)} d(g,r) \Big \}, \end{aligned}$$

where *d*(*g*, *r*) is the Euclidean distance between voxels and *g* and *r*, and $d_S(g, S)$ is the distance from voxel *g* to the closest point in the surface set *S*. In this way, MSD gives an average symmetric distance from the voxels of one surface to another (from the result to the reference and vice versa), while the HD gives the longest of all the distances from a voxel on one surface the closest voxel in the other. The HD95 metric takes the 95th percentile of the distances between the surface voxels, which diminishes the influence of outlier points.

**Figure 4:**
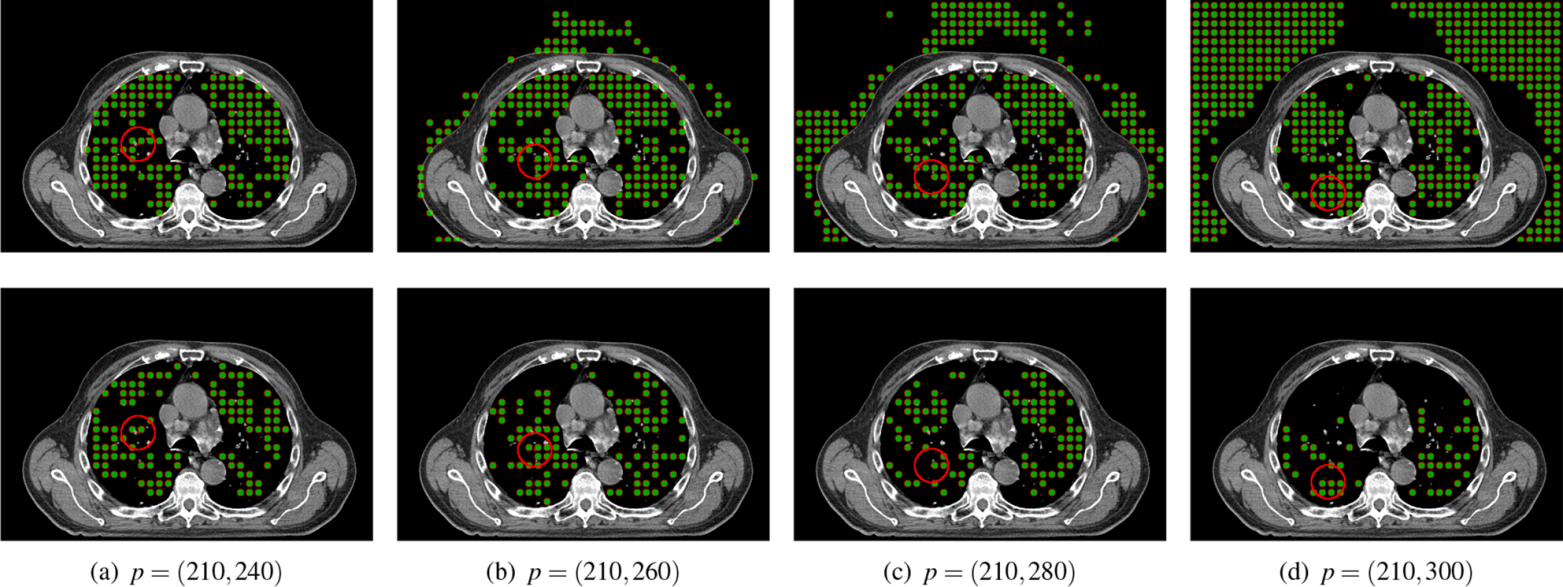
The influence of Gabor features on the stability of initialization relative to the position of the initial contour (section $z=65$ of the set Train-S1-001). Regions generated from four contour positions (centered at *p* and radius 20 voxels) using image intensity (top row) and Gabor features (bottom row).

**Figure 5:**
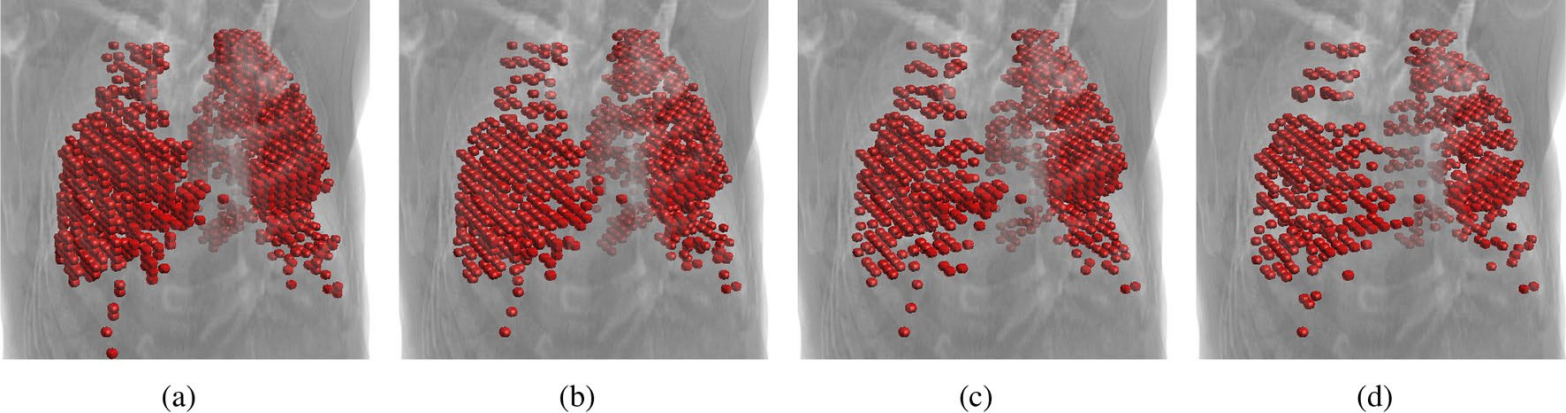
Initial surface regions generated with the spacing of (**a**) 2, (**b**) 3, (**c**) 4, and (**d**) 5 images (dataset Train-S1-001).

**Figure 6:**
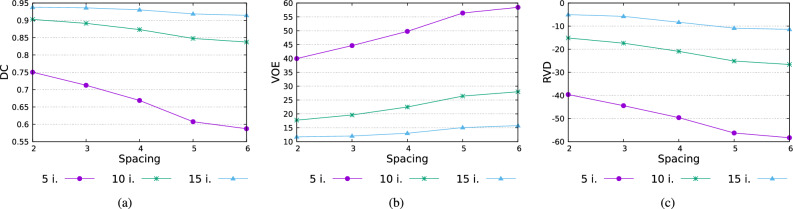
Quality of the segmentation results depending on the initialization image spacing (dataset Train-S1-001). Values of (**a**) DC, (**b**) VOE, and (**c**) RVD metrics after 5, 10, and 15 iterations of the active surface with different image spacings (from 2 to 6 images).

**Table 1:** Segmentation accuracy of the proposed method on the LCTSC online test datasets.

Dataset	Accuracy measure					Parameters		Iterations
	VOE	RVD	MSD	HD95	DC	$\varepsilon$	$\alpha$	
Test-S1-201	6.61	3.68	0.85	2	0.97	750	0.0125	30
Test-S1-202	6.81	3.27	0.93	3	0.96	750	0.01	55
Test-S1-203	3.52	0.4	0.77	3.5	0.98	750	0.0125	35
Test-S1-204	2.56	− 0.79	0.64	3	0.99	750	0.0125	30
Test-S2-201	7.62	2.46	0.83	2.5	0.96	750	0.0125	40
Test-S2-202	8.25	− 6.75	1.33	4	0.96	750	0.0125	30
Test-S2-203	15.15	10.58	1.52	5.5	0.92	750	0.0125	30
Test-S2-204	9.69	0.03	1.13	3	0.95	650	0.0125	25
Test-S3-201	9.53	2.78	1.14	4	0.95	650	0.02	45
Test-S3-202	2.98	− 1.43	0.84	4	0.98	750	0.0125	35
Test-S3-203	7.38	− 3.17	1.62	8	0.96	650	0.02	50
Test-S3-204	7.44	0.56	1.25	5	0.96	800	0.0125	50

Level set parameters and iterations required for each case also included.

**Figure 7:**
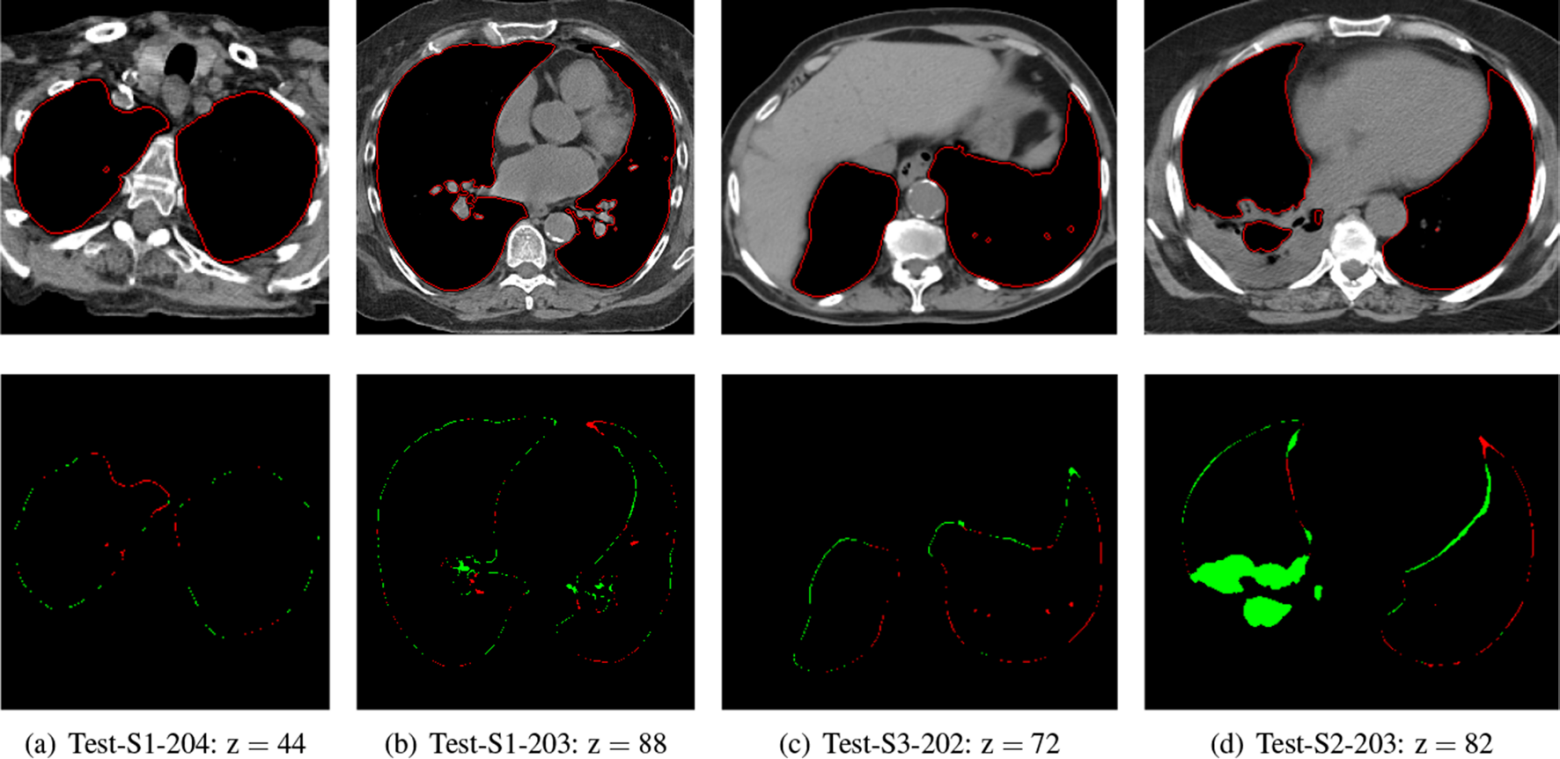
Example results of LCTSC images (dataset and image number provided): outlines of the final regions in the top row and segmentation errors (false positives in green and false negatives in red) in the bottom row.

### Results

The first example illustrates the working of the method on the Train-S1-001 LCTSC data set. Figure 3 presents the initial surface regions generated from three starting contours. The figure shows that it would be very difficult to obtain satisfying coverage of the lung volume with just one ellipse. The lung lobes exhibit clear differences in textural characteristics, but only three contours were sufficient to generate adequate surface regions.

The final result in Fig. 3e presents a successful segmentation (DC $=$ 0.97), performed with the 3D active surface model. The total time of the level set evolution was about 3.5 s, and the initial region generation was performed in 1.5 s. The results show an advantage of the 3D active surface over the 2D approach (see Fig. 3d). Although the texture-based 2D contour performs well on separate images, it processes the slices independently and the continuity of the final surface is harder to preserve. Additionally, automated segmentation in 2D required a constant set of parameters for all images, which ultimately was not well suited for all slices. Moreover, the 2D method required about 750 ms to process a single image, which gave about one minute of operation time. The rest of the presented results were obtained with the fully 3D active surface.

A demonstration of the influence of Gabor features on initialisation sensitivity is presented in Fig. 4. The top row contains initial surface regions generated for different positions of the start contour using the intensity of the image. The obtained regions show that a small change in the contour position can result in incorrect region selection. The utilisation of the Gabor features (bottom row) improves the robustness of the selection process and reduces the sensitivity to precise contour placement.

Another important factor of the initialization process is the image spacing. This parameter determines the number of images that will take part in the generation of initial areas. A spacing of *m* indicates that only every *m*-th image in the selected range will be processed (see “Initialisation and starting region selection”). The choice of image spacing has a big influence on the performance of the method. Initialization with a small gap naturally results in a larger starting area (see Fig. 5). This entails a faster evolution of the deformable model because a significant part of the target lung region is already covered at the start. On the other hand, a larger spacing speeds up the process of generating initial regions, which is a key step in making the solution interactive. In the conducted tests, it was assumed that the time delay between the modification of the starting contour by the operator and the generation of the starting regions should not take longer than 2 s. The effect of the spacing value on the segmentation quality is shown in Fig. 6, where DC, VOE, and RVD are shown for different values of the spacing and numbers of iterations of the active surface. The obtained data show that the differences in quality decrease after 15 iterations. Further tests showed that after 30-50 iterations the differences become negligible. The spacing from 2 to 4 images does not have a big impact on the segmentation precision, but it can have a real impact on the usability of the method. On the available platform, the generation time of the initial areas could be reduced by up to half by applying larger spacing (from 3.2 s for spacing 2 to 1.6 s for spacing 4). These differences would be more significant for lower-end graphics cards.

Table 1 contains the quality metrics of the results obtained on the LCTSC online test datasets, which were used in the final stage of the AAPM 2017 Grand Challenge to evaluate multiple auto-segmentation methods. The sets contain mostly typical lung anatomy with small and medium-sized pathological changes (eg. sets Test-S1-201 and Test-S1-204), as well as large tumours and more abnormal lung structures (Test-S2-203). More problematic cases with extreme pathology or acquisition issues were not included in the test sets.

The metrics (DC, MSD, and HD95) were also utilised in the AAPM Challenge. In the case of lung segmentation, the AAMP methods achieved, on average, DC $=$ 0.97 (ranging from 0.95 to 0.98), MSD $=$ 1.23, and HD95 $=$ 4.8. The results of the presented algorithm are generally on par with the methods competing in the Challenge, with DC $\ge$ 0.96 in most of the selected cases and comparable values of MSD and HD95.

It should be noted that the AAPM Challenge was aimed at automatic segmentation and contained many deep learning-based approaches. Most of the methods used convolutional networks, often based on the U-Net architecture^41^. Multi-atlas schemes^19^ were also utilised by some participants. Many of these methods required a long training stage, which often took multiple days to complete^20^. In contrast, the proposed semi-automatic scheme requires some manual initialisation, but the final computations are completed within seconds.

The last example in Fig. 7 shows a sample of typical results on original 2D images. The error images in the figure indicate that the segmentation errors are typically located on the outer surface of the lungs. This type of slight under- or over-segmentation could be resolved in post-processing (e.g. by morphological erosion or dilation). The hilar region is also an area of possible ambiguities, due to the presence of bronchi, blood vessels, and nerves. Figure 7d shows a slice of the Test-S1-203 dataset, which contains the most extreme pathological changes and posed the most problems for the proposed approach, as well as for the AAPM methods.

## Discussion

The proposed method was evaluated in real data sets and the results were compared with the performance of several other segmentation methods from the AAPM Challenge. The obtained segmentations were acceptable in most cases, taking into account a certain ambiguity and iter-rater variability typical for medical imaging. One of the tested datasets (Test-S2-203) contains substantial pathological changes in the right lung. Both AAPM methods and the presented approach gave worse results for this set in contrast to the rest of the studies. This example illustrates a limitation of per-voxel segmentation methods. Although the proposed method technically segmented the tissues according to the designed texture-based similarity criterion, the final decision to exclude the entire region was made according to the expertise of the radiologists performing the ground truth contouring. The excluded area was non-homogenous even in regard to the texture and contained different types of tissue. This indicates that particularly difficult cases can pose problems for methods that rely on raw imaging data. On the other hand, this example illustrates the need for interactive methods that are well-suited for manual refinement in such atypical cases.

When it comes to interactive methods, one of the most important factors is the input of the operator which is necessary to obtain appropriate results. The thoracic CT studies can contain over 100 slices with lung tissue. The proposed method requires a small initial input, where usually only 3 slices have to be marked to obtain the initial regions. Due to the utilisation of a probabilistic texture-based scheme, a single image can be processed in sub-second (400–500 ms) time without parameter manipulation. The default initialisation parameters were sufficient in all cases and only a few of the test datasets required an addition of a region of interest (ROI) to limit the initialisation, but even with this operation, the operator labour is small. The next segmentation stages are also very efficient and can give final results for both lungs in less than 5 s total. This performance comes from the GPU acceleration, as well as from the algorithm itself because a large part of the target regions can be found before the main level set evolution stage.

The final tuning of the parameters in the last stage can be beneficial for the quality of the results. Very fast execution makes this process less cumbersome. During validation on the LCTSC test data, the default values of the level set method were suitable for most of the sets and had to be slightly tuned only in 5 cases (see Tab. 1). The $\varepsilon$ and $\alpha$ parameters did not exhibit a high sensitivity to change and clear differences in segmentation results could be observed only for their more extreme values (e.g. for $\varepsilon$ below 500 and above 900). The active surface needed typically 30 to 40 iterations to converge.

The proposed method greatly benefits from GPU hardware acceleration. The presented results were obtained using an “enthusiast-grade” Nvidia Titan Xp card, launched in 2017. With the progress in the GPU computational power over the years, even mid-range and mobile graphics cards have computational and memory capabilities adequate for interactive segmentation of typical CT data sets. The presented solution does not require any high-end hardware and can be deployed on a wide variety of workstations due to ubiquitous OpenCL support.

## Conclusion

In this paper, a GPU-accelerated level set method for the segmentation of lung CT volumes is proposed. The method takes advantage of the computational power of modern graphics processors to provide a semi-automatic tool for near-interactive segmentation. The performed experiments show that the method is able to successfully segment real medical datasets in a matter of seconds, while the texture-based approach is beneficial for the quality of the results.

The current version of the method could be improved in multiple ways. One obvious direction is to develop an automatic initialisation scheme that would eliminate (or at least reduce) the need for the operator’s work. On the other hand, the algorithm can go in the direction of a fully interactive segmentation^26^, since the process of manual initialisation (slice-by-slice vs entire volume) and level set optimisations can be further enhanced. Finally, a more generic feature selection can be applied to eliminate the need for problem-specific feature banks and open possibilities of application in other tasks.

## Data Availability

The CT datasets used in this research are provided by The Cancer Imaging Archive^36^ under the CC BY 3.0 license. The datasets are publicly available at: https://wiki.cancerimagingarchive.net/pages/viewpage.action?pageId=24284539. The data was used according to the TCIA Data Usage Policy and Restrictions and all research was carried out in accordance with relevant guidelines and regulations.
